# Effectiveness and safety of theta burst stimulation for Parkinson's disease: an updated systematic review and meta-analysis of randomized parallel-group controlled trials

**DOI:** 10.3389/fneur.2026.1915632

**Published:** 2026-09-23

**Authors:** Qing Xu, Shuqin Hao, Yebo Wang, Wenjie Xi, Yanmin Li, Yahui Wang

**Affiliations:** 1 Department of Rehabilitation Medicine, First Hospital of Hebei Medical University, Shijiazhuang, Hebei, China; 2 Health Management Center, First Hospital of Hebei Medical University, Shijiazhuang, Hebei, China; 3 Department of Neurology, Handan Central Hospital, Handan, Hebei, China; 4 Department of Parkinson's Disease, First Hospital of Hebei Medical University, Shijiazhuang, Hebei, China

**Keywords:** cognition, depression, meta-analysis, motor, Parkinson's disease, theta burst stimulation

## Abstract

**Background:**

Parkinson's disease (PD) is a progressive neurodegenerative disorder characterized by motor and non-motor impairments that are often incompletely controlled by pharmacological treatment. Theta burst stimulation (TBS), including transcranial and transspinal approaches, has emerged as a potential neuromodulation strategy for symptom management in PD. This updated systematic review and meta-analysis evaluated the efficacy and safety of TBS for motor and non-motor outcomes in individuals with PD.

**Methods:**

PubMed, Embase, Web of Science, the Cochrane Library, and ClinicalTrials.gov were searched from inception to 28 July 2026. Randomized parallel-group controlled trials comparing active TBS with sham stimulation in individuals with PD were included. Two reviewers independently performed study selection, data extraction, and risk of bias assessment using the Cochrane Risk of Bias 2 tool. Random-effects meta-analyses were conducted, and certainty of evidence was assessed using the Grading of Recommendations Assessment, Development and Evaluation (GRADE) framework.

**Results:**

Twelve randomized parallel-group controlled trials including 388 participants were included. Active transcranial TBS significantly improved motor symptoms in the OFF-medication state (*SMD* = −0.43; 95% *CI*, −0.78 to −0.09; *P* = 0.01; *I*^2^ = 3%; high-certainty evidence), whereas no significant benefit was observed in the ON-medication state or at 1-month follow-up. Active transcranial TBS also reduced depressive symptom scores (*SMD* = −0.60; 95% *CI*, −1.07 to −0.12; *P* = 0.01; *I*^2^ = 36%; high-certainty evidence). Moderate-certainty evidence indicated potential improvements in cognitive outcomes following transcranial TBS immediately after treatment and at 3-month follow-up, as well as potential motor benefits of transspinal TBS immediately after treatment in the ON-medication state. These findings should be interpreted cautiously given the small number of trials and participants and heterogeneity in anatomical stimulation targets and stimulation protocols. No serious stimulation-related adverse events were reported.

**Conclusion:**

Transcranial TBS may improve short-term motor symptoms in the OFF-medication state and reduce depressive symptom scores. Cognitive benefits following transcranial TBS and the potential motor effects of transspinal TBS warrant cautious interpretation. Larger trials with standardized stimulation protocols and longer follow-up are needed to establish the durability and clinical relevance of these effects.

**Systematic review registration:**

https://www.crd.york.ac.uk/prospero/search, identifier CRD420261285544.

## Introduction

Parkinson's disease (PD) is the second most common neurodegenerative disorder worldwide and is characterized by progressive motor and non-motor impairments that substantially reduce functional independence and quality of life (1–4). Although dopaminergic therapies remain the mainstay of treatment, many symptoms—including gait impairment, cognitive dysfunction, and depression—often respond incompletely to medication or worsen over time (5). This highlights the need for complementary interventions to better manage motor and non-motor symptoms in individuals with Parkinson's disease.

Repetitive transcranial magnetic stimulation (rTMS) is a non-invasive neuromodulation technique that may influence both motor and non-motor symptoms in Parkinson's disease through modulation of distributed neural networks (6–9). Current clinical guidelines support the probable efficacy of high-frequency rTMS for motor symptoms and depression in PD (10). Theta burst stimulation (TBS) is a patterned form of rTMS that mimics the brain's natural theta rhythms to efficiently induce synaptic plasticity (11). Its much shorter stimulation duration (typically seconds to a few minutes) compared to conventional rTMS has made it an increasingly applied tool in neurology, psychiatry, and neurorehabilitation (12–15). More recently, stimulation approaches have expanded beyond conventional brain-targeted approaches to include transspinal magnetic stimulation, with preliminary evidence suggesting potential benefits for both motor and non-motor symptoms in PD (16, 17).

Despite growing interest in the use of TBS for Parkinson's disease, the available evidence remains limited and continues to evolve. A previous systematic review and meta-analysis summarized the effects of TBS on motor and non-motor dysfunction in PD based on studies published up to 2021 (3). Since then, several additional randomized controlled trials have been conducted, expanding the evidence base and introducing novel stimulation approaches, including transspinal TBS protocols. Consequently, an updated synthesis of the available evidence is warranted to provide a more comprehensive evaluation of the therapeutic effects of TBS in PD.

Therefore, we conducted a systematic review and meta-analysis of randomized controlled trials to evaluate the effects of TBS on motor and non-motor outcomes in individuals with Parkinson's disease. By incorporating recently published studies and expanding the evidence base to include both transcranial and transspinal TBS approaches, this review aims to provide an updated evaluation of the efficacy and safety of TBS and to identify priorities for future research.

## Methods

### Search strategy

This systematic review and meta-analysis followed the PRISMA 2020 statement. Electronic searches were conducted in PubMed, Embase, Web of Science, the Cochrane Library, and ClinicalTrials.gov from inception to 28 July 2026. Reference lists of eligible articles were also manually examined to identify additional studies. Search strategies incorporated a combination of controlled vocabulary and free-text terms related to Parkinson's disease and theta burst stimulation. Filters for randomized trials were applied using publication type and title/abstract terms. Randomized crossover trials were not specifically excluded by the search strategy and were subsequently excluded during full-text assessment because the eligibility criteria were restricted to randomized parallel-group controlled trials. The complete search strategies, including the exact search strings, search limits, and final search dates for each database, are provided in Supplementary Table S1. The search yielded 1,712 records concerning theta burst–based magnetic stimulation interventions in PD. As this investigation synthesized published data only, ethical approval and informed consent were not required. PROSPERO registration: CRD420261285544.

### Eligibility criteria

Randomized controlled trials examining the effects of TBS on motor and/or non-motor outcomes in individuals with Parkinson's disease were considered eligible. Studies were required to satisfy predefined PICOS criteria: participants were individuals diagnosed with Parkinson's disease (P); Interventions consisted of active transcranial or transspinal TBS, with no restrictions on stimulation intensity, number of sessions, or treatment duration (I); comparators were sham or placebo interventions designed to mimic TBS without delivering an effective magnetic field (C); outcomes included clinical assessments of motor and/or non-motor symptoms (O); and study design was restricted to randomized parallel-group controlled trials (S).

### Data extraction

Two reviewers independently screened titles and abstracts to identify potentially relevant studies. Full-text articles were obtained when eligibility could not be determined at the initial screening stage. For studies lacking data necessary for quantitative synthesis, corresponding authors were contacted to obtain additional information. Any disagreements were resolved by discussion, with arbitration by a third reviewer when required. Data were extracted independently using a standardized form and included: first author, year of publication, participant characteristics (age, sex, disease duration, Hoehn and Yahr stage, levodopa equivalent daily dose), sample size, outcome measures, assessment time points, adverse events, TBS intervention parameters, and methodological quality.

### Risk of bias assessment

Risk of bias was assessed using the Cochrane Risk of Bias 2 (RoB 2) tool for randomized trials. The assessment was conducted at the outcome level rather than at the study level, considering specific outcome measures and assessment time points reported in each trial. The RoB 2 tool evaluates five domains: randomization process (D1), deviations from intended interventions (D2), missing outcome data (D3), measurement of the outcome (D4), and selection of the reported result (D5). Each domain was rated as “low risk of bias,” “some concerns,” or “high risk of bias,” and an overall judgment was assigned for each assessed outcome. The assessments were performed according to the effect of assignment to intervention (intention-to-treat effect), reflecting the impact of treatment allocation rather than adherence to the intervention.

### Data synthesis and statistical analysis

When two or more studies reported comparable outcomes, quantitative synthesis was performed using Review Manager (RevMan, version 5.4, The Cochrane Collaboration, London, United Kingdom). For continuous outcomes measured using the same scale, pooled effects were expressed as mean differences (MDs) with 95% confidence intervals (CIs), whereas standardized mean differences (SMDs) were used when different measurement scales were applied.

Treatment effects were calculated using change-from-baseline scores when baseline and post-intervention data were available. Mean changes were calculated as the difference between post-intervention and baseline values. When standard deviations (SDs) of change scores were not directly reported, they were estimated using the following formula:


$$\begin{array}{l} SD_{change}=\sqrt{SD_{baseline}^{2}+SD_{post}^{2}-2r\times SD_{baseline}\times SD_{post}} \end{array}$$

A correlation coefficient (*r*) of 0.5 was assumed because the correlation between baseline and post-intervention measurements was not reported in the included studies, according to the Cochrane Handbook recommendations (18). Standard errors of the mean (SEM) were converted to SDs using $SD=SEM\times \sqrt{N}$ (19). For outcomes reported as medians with ranges or interquartile ranges, means and SDs were estimated using the methods described by Hozo et al. (20). When only ranges were available, the midpoint was used to approximate the mean (21).

The overall sample size was defined based on participants included in eligible active TBS vs. sham stimulation comparisons. For multi-arm trials, only intervention and comparator arms directly addressing the predefined review question were included in the quantitative synthesis. Arms involving additional co-interventions or alternative stimulation protocols were extracted for descriptive purposes but were not included in the primary meta-analyses. Outcomes reported under different medication states (ON or OFF medication) were analyzed separately to account for potential clinical heterogeneity. Outcome-specific sample sizes contributing to each meta-analysis are presented in the corresponding forest plots and GRADE tables.

All pooled analyses were conducted using a random-effects model (DerSimonian–Laird method) because of anticipated clinical and methodological heterogeneity across studies (22, 23). Results were presented as forest plots. Heterogeneity was assessed using the chi-square (χ^2^) test and quantified using the *I*^2^ statistic, with *p* < 0.10 or *I*^2^ > 50% indicating substantial heterogeneity (24–26). Sensitivity analyses were performed to evaluate the robustness of pooled estimates. Publication bias was not assessed when fewer than 10 studies were available for an outcome.

### Certainty of evidence

The certainty of evidence was evaluated using the Grading of Recommendations, Assessment, Development and Evaluation (GRADE) framework. The five domains of risk of bias, inconsistency, indirectness, imprecision, and publication bias were considered.

## Results

### Search results

The study selection process is illustrated in the PRISMA flow diagram (Figure 1). A total of 1,712 records were identified through searches of the prespecified databases. After removal of duplicate records and records not representing clinical trials, 856 records were excluded. During title and abstract screening, a further 703 records were excluded because they focused on unrelated topics or involved different stimulation modalities. Subsequently, 153 reports were sought for retrieval, of which 10 could not be retrieved. The remaining 143 reports were assessed for eligibility. Of these, 23 conference abstracts and 85 research protocols were excluded. Among the remaining full-text reports, six were excluded because of ineligible stimulation types, six were non-randomized studies, three were randomized trials without a sham-control group, and eight were randomized crossover trials. Key studies excluded for reasons relevant to the study-design and comparator criteria are summarized in Supplementary Table 2. Finally, 12 studies (17, 27–37) were included in the meta-analysis.

**Figure 1:**
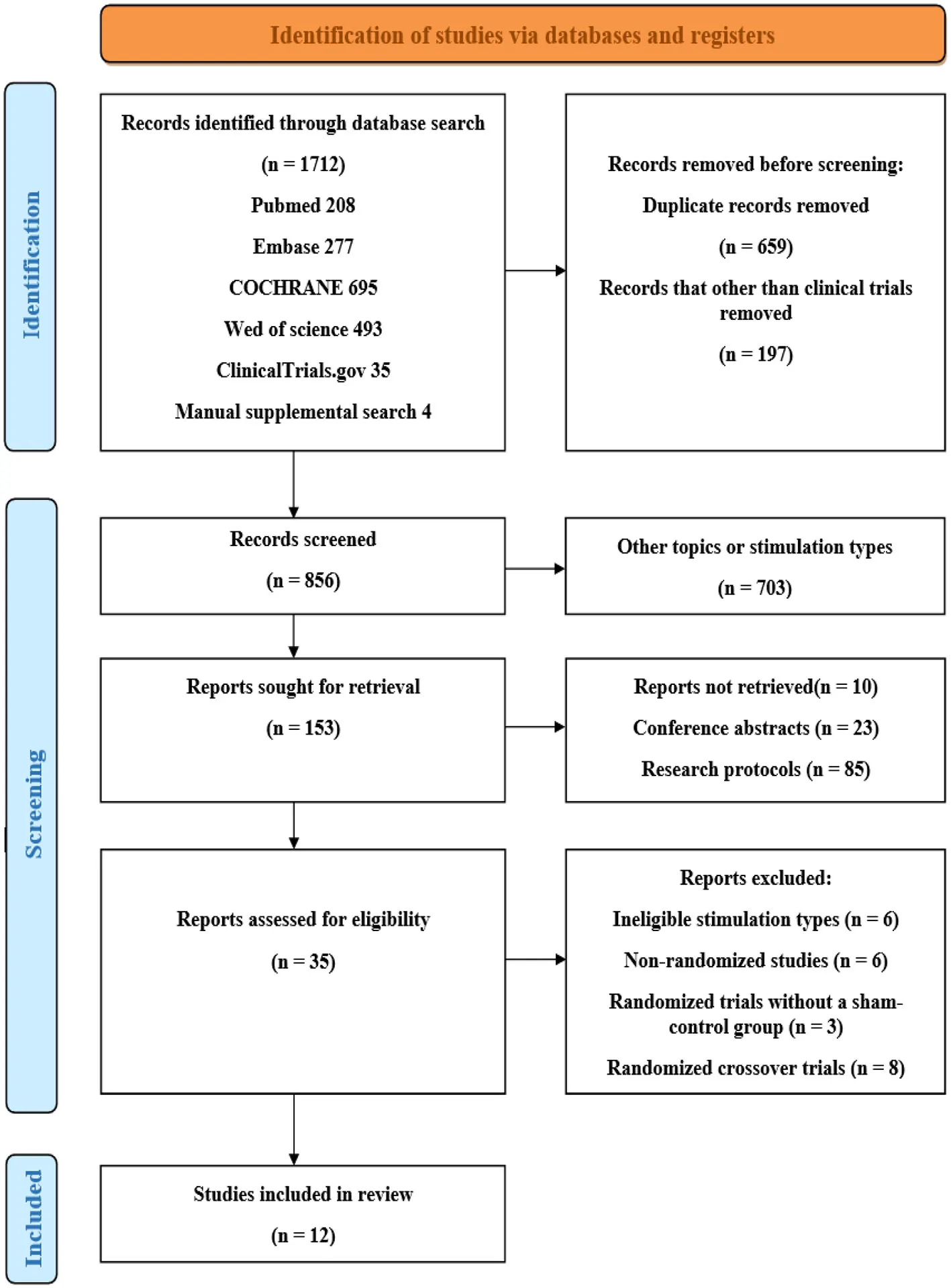
Study selection.

### Study characteristics

Among the included trials, 10 studies evaluated transcranial TBS (27–36), whereas two studies investigated transspinal TBS (17, 37). Two studies (32, 35) included multiple intervention arms. All studies involved multiple stimulation sessions. Detailed characteristics of the included studies are provided in Table 1, while TBS intervention parameters are summarized in Table 2.

**Table 1:** Demographic and clinical characteristics of included studies.

Study	Age, y	Sample size	PD duration, y	Sex (M/F)	HandY stage	LEDD, mg	Assessment time	Outcome measures (Clinical assessment)
Kotsoev et al. (27)	E: 65,5 [60,0;72,5] C: 64,5 [57,0;72,8]	E: 12 C: 12	E: 8,5 [4,0; 10,0] C: 6,0 [4,0; 9,0]	E: 2/10 C: 6/6	E: II 4; III 8 C: II 6; III 6	E: 923,8 [828,1;1,130,0] C: 650,0 [531,9;888,1]	Baseline, 1st day, 1st month, 3rd month	MDS-UPDRS III, BBS, 10WMT, Postural instability
Benninger et al. (28)	E: 62.1 ± 6.9 C: 65.6 ± 9.0	E: 13 C: 13	E: 10.8 ± 7.1 C: 6.5 ± 3.4	E: 11/2 C: 7/6	E: 2.6 ± 0.2 C: 2.5 ± 0.1	E: 1,180.9 ± 662.4 C: 732.3 ± 344.8	Baseline, 1st day, 1st month	UPDRS, 10MWT, Bradykinesia, FAB, BDI, SF-12v2™, SRTT-8
Lang et al. (29)	E: 68.43 ± 8.4 C: 68.76 ± 8.3	E: 21 C: 20	E: 5.95 ± 4.8 C: 4.80 ± 4.0	E: 14/7 C: 13/7	NA	E: 911.95 ± 522.2 C: 930.10 ± 396.2	Baseline, 1st day, 1st month	Primary: Five cognitive domains Secondary: UPDRS-III, BDI-II, BAI
Ji et al. (30)	E: 61.7 (1.57) C: 60.2 (1.97)	E: 22 C: 20	E: 4.3 (0.52) C: 5.3 (0.83)	E: 14/8 C: 14/6	E: 1.6 (0.12) C: 1.7 (0.11)	E: 485.2 (58.08) C: 460.5 (68.04)	Baseline, 1st week, 2nd week	Primary: UPDRS-III (2 weeks) Secondary: UPDRS-III (1 week), NMSS, TUG, 20WMT
Koch et al. (31)	64.7 ± 6.9	E: 10 C: 10	10.4 ± 4.3	NA	NA	NA	Baseline, 2nd week, 4th week, 6th week	UPDRS-III, Global CAPSIT dyskinesia scale scores
Cheng et al. (32)	E_1_ (TBS-VR): 71.4 ± 8.5 E_2_ (TBS): 71.6 ± 5.1 C: 73.9 ± 6.9	E_1_:13 E_2_:11 C: 16	NA	E_1_: 7/6 E_2_:6/5 C: 11/5	E_1_:2.2 ± 0.9 E_2_:3.0 ± 1.2 C: 2.5 ± 1.0	E_1_:635.3 ± 335.1 E_2_:573.6 ± 272.7 C: 681 ± 324.6	Baseline, 1st day, 3rd month	RBANS, MoCA, BDI
He et al. (33)	E: 70.0 ± 6.3 C: 74.8 ± 6.9	E: 20 C: 15	E: 2.7 ± 1.5 C: 2.5 ± 1.1	E: 13/7 C: 10/5	E: 2.7 ± 1.5 C: 2.5 ± 1.1	E: 604.4 ± 295.2 C: 64 7.9 ± 323.5	Baseline, Immediately, 3rd month	RBANS, MoCA
Trung et al. (34)	E: 71.3 ± 7.3 C: 67.3 ± 5.2	E: 14 C: 14	E: 10.39 ± 6.7 C: 6.25 ± 3.0	E: 8/6 C: 11/3	1–3	E: 934 ±593 C: 911 ± 699	Baseline, 1st day, 10th day, 30th day	Primary: Neuropsychological Test battery according to cognitive domains Secondary: SETS, BDI, BAI, AES, PDQ-39, UPDRS-III, MoCA
Hou et al. (35)	E_1_ (TBS): 69.9 ± 4.7 E_2_ (HF-rTMS): 69.9 ± 6.2 C: 70.5 ± 5.3	E_1_:18 E_2_:18 C: 18	E_1_: 7.3 [4.0,9.0] E_2_: 4.3 [1.0,7.5] C: 4.5 [2.0,8.5]	E_1_:11/7 E_2_:11/7 C: 7/11	E_1_:2 [2,3] E_2_: 2 [2,3] C: 2 [2,3]	E_1_:750 [375,750] E_2_:750 [500,750] C: 750 [500,750]	Baseline, 1st week, 2nd week, 6th week	Primary: HAMD-24 (2 weeks) Secondary: MDS-UPDRS III, HAMA-14, PDQ-8
Zhang et al. (36)	E: 65 [60,66.5] C: 63 [58.5,66.5]	E: 25 C: 25	E: 6 [5,8] C: 7 [5,9]	E: 10/15 C: 7/18	E: I 1; II 15; III 9 C: I 0; II 17; III 8	E: 466.5 [387.25,576.75] C: 512.5 [408.00,576.75]	Baseline, 2nd week, 4th week, 12th week, 24th week	Primary: MDS-UPDRS III^b^ (2 weeks) Secondary: MDS-UPDRS III^a^ (2 weeks); PDQ-39 (4 weeks, 12 weeks, 24 weeks) Exploratory: 6 MWT, GFQ, Gait Analysis^b^
Menezes et al. (37)	E: 68.56 ± 6.4 C: 70.3 ± 6.3	E: 16 C: 17	E: 9.0 ±5.16 C: 7.0 ± 8.75	NA	NA	NA	Baseline, immediately, 1st week, 4th week	Primary: TUG Secondary: BESTest, UPDRS-III, dual-task TUG, FAB, percentage of FOG, FOG score, NFoG-Q, PDQ-39
Lapa et al. (17)	E: 61.9 ± 10.3 C: 61.4 ± 9.2	E: 13 C: 13	E: 10.9 ± 5.2 C: 9.3 ± 7.5	E: 5/13 C: 9/13	E: 2.46 ± 1.18 C: 2.15 ± 0.89	E: 847.8 ± 425.7 C: 936.4 ± 468.7	Baseline, 8th week	Primary: NRS Secondary : PD-PCS, HADS-A, HADS-D, EuroQol-5, UPDRS-III, brief pain inventory, Likert scale

Mean ± SD/mean (SE)/median [Q_1_, Q_3_]. SD, standard deviation; SE, standard error; Q_1_, first quartile; Q_3_, third quartile; C, control group; E, experimental group; PD, Parkinson's Disease; HandY, Hoehn and Yahr scale; TBS, theta-burst stimulation; TBS-VR, theta-burst stimulation combined with virtual reality; HF-rTMS, high-frequency repetitive transcranial magnetic stimulation; NA, not available; MDS, Movement Disorder Society; UPDRS, Unified Parkinson's Disease Rating Scale ( part III, motor examination); BBS, Berg balance scale; 10MWT, 10 Meter Walk Test; FAB, Frontal Assessment Battery; BDI, Beck Depression Inventory; SF-12v2™,short form of a health survey; SRTT, Serial Reaction Time Task; BAI, Beck Anxiety Inventory; NMSS, Non-Motor Syndrome Scale; TUG, Timed Up and Go; 20MWT, 20 Meter Walk Test; RBANS, Repeatable Battery for the Assessment of Neuropsychological Status; MoCA, Montreal Cognitive Assessment; SETS, Stanford Expectations of Treatment Scale; AES, Apathy Evaluation Scale; PDQ-39, Parkinson's Disease Questionnaire-39; HAMD-24, 24-item Hamilton Depression Rating Scale; HAMA-14, 14-item Hamilton Anxiety Rating Scale; PDQ-8, 8-item Parkinson's Disease Questionnaire, 6 MWT, 6-Minute Walk Test; GFQ, Gait and Falls Questionnaire; BESTest, Balance Evaluation System Test; FOG, freezing of gait; NFOG-Q, New Freezing of Gait Questionnaire; NRS, Numerical Rating Scale; PD-PCS, Parkinson's Disease Pain Classification System; HADS-A, Hospital Anxiety and Depression Scale-Anxiety; HADS-D, Hospital Anxiety and Depression Scale-Depression; EuroQol-5, EuroQol Five-Dimensional Questionnaire.

^a^“On” anti-Parkinsonism medicine.

^b^“Off” anti-Parkinsonism medicine.

**Table 2:** Stimulation parameters of TBS protocols included in the meta-analysis.

Study	Treatment protocol	Frequency	Intensity	Coil-type	Stimulation target/site	Continuous/ discontinuous days	Sessions/ duration	Pulses/ session
Kotsoev et al. (27)	iTBS	50 Hz	80%AMT	F8	M1 (bilateral)	Continuous (10 days)	10/10 days	600
Benninger et al. (28)	iTBS	50 Hz	80%AMT	C	DLPFC + M1 (bilateral)	Continuous (4 days)	8/2 weeks	600
Lang et al. (29)	iTBS	50 Hz	80%AMT	F8	DLPFC (left)	Continuous (6 days)	6/1 week	600
Ji et al. (30)	cTBS	50 Hz	80%AMT	F8	SMA (left)	Continuous (14 days)	42/14 days	600
Koch et al. (31)	cTBS	50 Hz	80%AMT	F8	Cerebellar (bilateral)	Continuous (10 days)	10/2 weeks	600
Cheng et al. (32)	iTBS	50 Hz	90%RMT	F8	DLPFC (left)	Continuous (10 days)	10/2 weeks	600
He et al. (33)	iTBS	50 Hz	100%RMT	F8	DLPFC (left)	Continuous (10 days)	10/2 weeks	600
Trung et al. (34)	iTBS	50 Hz	80%AMT	F8	DLPFC (left)	Discontinuous (1/1–2 days)	6/1 week (within)	600
Hou et al. (35)	iTBS	50 Hz	70%RMT	F8	DLPFC (left)	Continuous (10 days)	10/2 weeks	600
Zhang et al. (36)	iTBS	50 Hz	80%RMT	F8	M1 (bilateral)	Continuous (10 days)	20/2 weeks	600
Menezes et al. (37)	iTBS	50 Hz	100%TsMT	C	third thoracic vertebra at midline	Continuous (5 days)	5/5 days	1,200
Lapa et al. (17)	cTBS	50 Hz	100%TsMT	C	seventh cervical vertebra at midline	1st week: Continuous (5 days) 2nd−8th week: Discontinuous (2/1 week)	1st week: 5/5 days 2nd−8th week: 14/7 weeks	1,200

iTBS, intermittent theta-burst stimulation; cTBS, continuous theta-burst stimulation; AMT, active motor threshold; RMT, resting motor threshold; TsMT; trans-spinal motor threshold; F8, figure-of-eight coil; C, circular coil; M1, primary motor cortex; DLPFC, dorsolateral pre-frontal cortex; SMA, supplementary motor area.

### Risk of bias assessment

Risk-of-bias assessments were performed using the RoB 2 tool for each outcome at each assessment time point (Supplementary Figures S1–S6). Overall, the judgments were largely consistent across outcomes. No outcome assessment was judged as having a high risk of bias for the randomization process (D1) or selection of the reported result (D5). Most assessments were rated as low risk for deviations from intended interventions (D2), missing outcome data (D3), and measurement of the outcome (D4), although some concerns and high-risk judgments were identified for D3 in a small number of outcome assessments. Some concerns for D1 mainly reflected insufficient reporting of allocation concealment. One gait outcome was rated as having some concerns for D3 because one participant was unable to perform the 10-m walk test during the OFF medication state owing to severe painful foot dystonia. In the same study, one motor outcome at the 1-month follow-up was judged as having a high risk of bias for D3 because of substantial missing outcome data resulting from participant withdrawal or the need for therapy adjustment (27). One outcome assessment from Trung et al. (34) was rated as having some concerns for D4 because blinding of outcome assessors was not clearly reported. Some concerns for D5 mainly reflected the absence of a prospectively registered protocol or prespecified statistical analysis plan.

### Effects of TBS on clinical outcomes

#### Effects of transcranial TBS on motor symptoms

##### Immediate post-treatment

Seven studies evaluating transcranial TBS reported immediate post-treatment motoroutcomes assessed using the Unified Parkinson's Disease Rating Scale part III (UPDRS-III) (28, 30, 31, 34) or Movement Disorder Society-UPDRS part III (MDS-UPDRS-III) (27, 35, 36). Intermittent TBS (iTBS) was delivered to the bilateral primary motor cortex (M1) (27, 36), the left dorsolateral pre-frontal cortex (DLPFC) (34, 35), or the bilateral DLPFC and M1 (28), whereas continuous TBS (cTBS) was delivered to the left supplementary motor area (SMA) (30) or bilateral cerebellum (31). Meta-analyses were performed using change-from-baseline scores. Because medication status may influence motor performance, outcomes were analyzed separately according to ON and OFF medication states.

In the OFF medication state (Figure 2A), the pooled estimate favored active transcranial TBS over sham stimulation for motor symptoms (*SMD* = −0.43; 95% *CI*, −0.78 to −0.09; *Z* = 2.48; *P* = 0.01), indicating a moderate standardized effect size for the reduction of motor symptom severity (38). No evidence of statistical heterogeneity was detected (χ^2^ = 3.11; *P* = 0.38; *I*^2^ = 3%). In the ON medication state (Figure 2B), the pooled estimate did not reach statistical significance between active transcranial TBS and sham stimulation (*SMD* = −0.07; 95% *CI*, −0.36 to 0.22; *Z* = 0.50; *P* = 0.62). No evidence of statistical heterogeneity was detected (χ^2^ = 1.37; *P* = 0.93; *I*^2^ = 0%).

**Figure 2:**
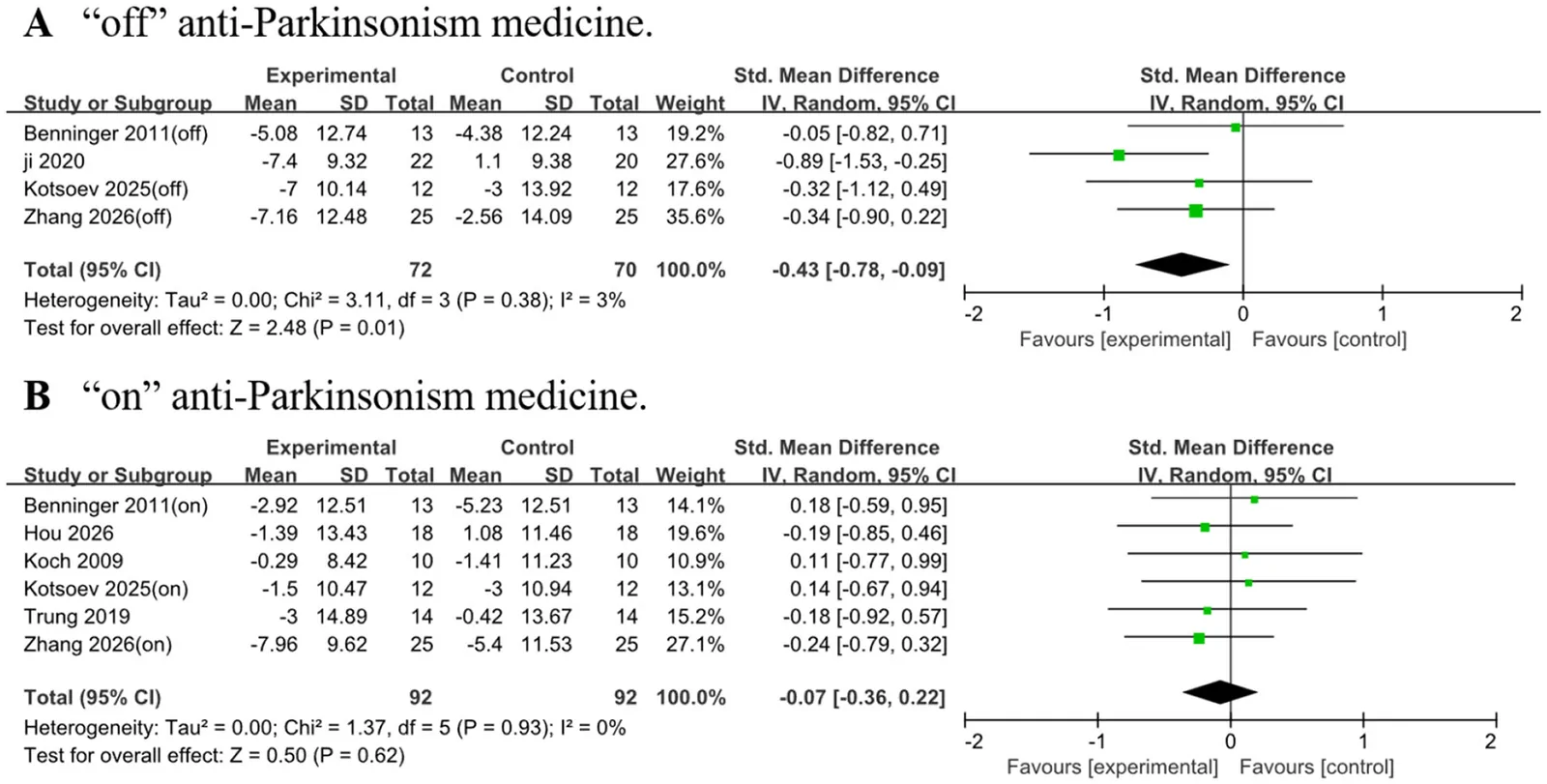
Forest plots of the effects of transcranial TBS on immediate post-treatment motor symptoms assessed by UPDRS-III/MDS-UPDRS-III. **(A)** OFF medication state; **(B)** ON medication state.

Subgroup analyses were further conducted according to stimulation pattern and stimulation target (Figure 3). For stimulation pattern, cTBS was not quantitatively pooled because only one study was available in each medication state (30, 31). In the OFF medication state (Figure 3A), three studies evaluating iTBS were included, and the pooled estimate did not reach statistical significance (*SMD* = −0.26; 95% *CI*, −0.65 to 0.13; *Z* = 1.29; *P* = 0.20), with no evidence of statistical heterogeneity (χ^2^ = 0.37; *P* = 0.83; *I*^2^ = 0%). In the ON medication state (Figure 3B), five studies evaluating iTBS were included, and the pooled estimate also did not reach statistical significance (*SMD* = −0.10; 95% *CI*, −0.40 to 0.21; *Z* = 0.62; *P* = 0.54), with no evidence of statistical heterogeneity (χ^2^ = 1.19; *P* = 0.88; *I*^2^ = 0%).

**Figure 3:**
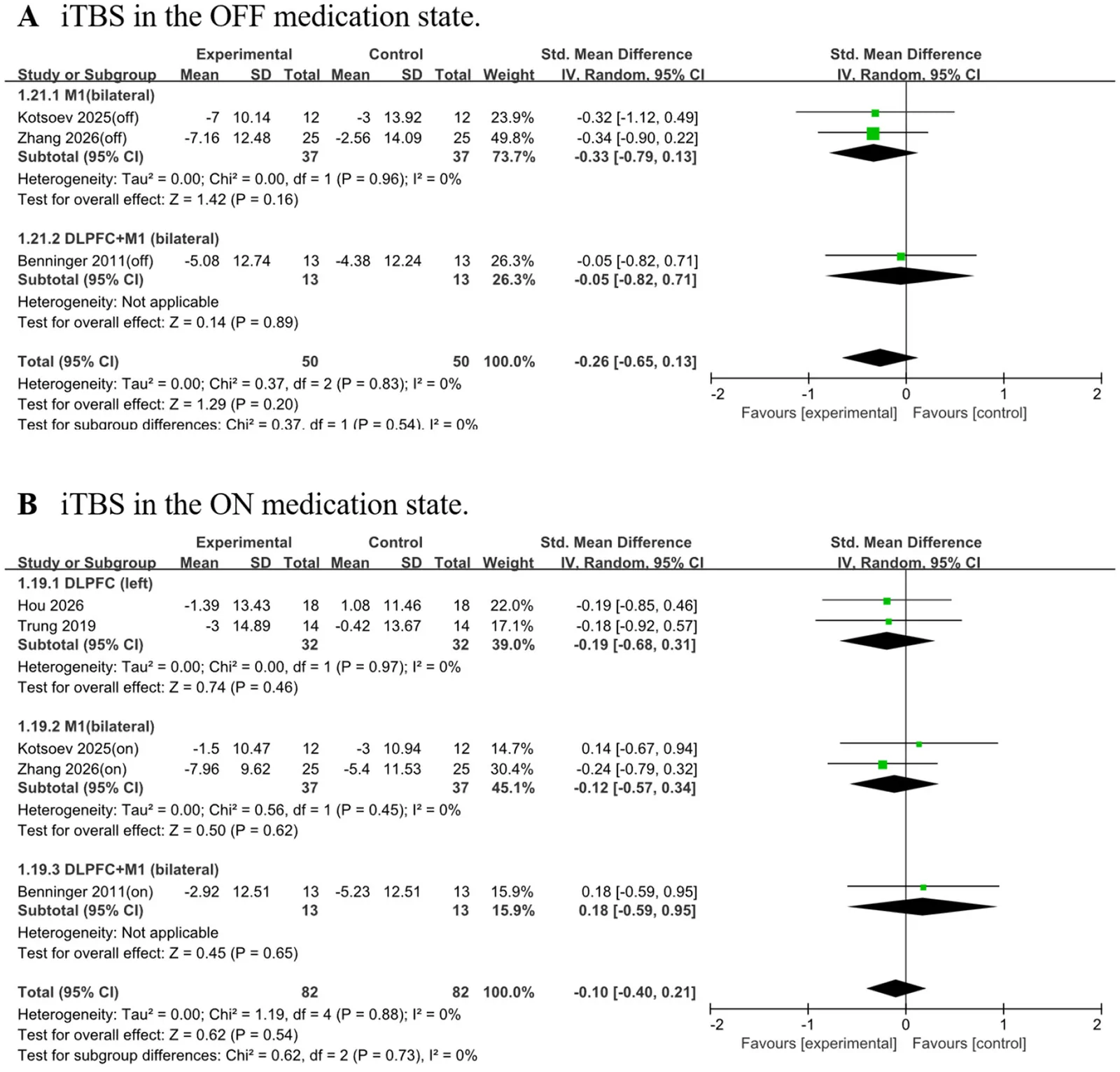
Subgroup analyses of immediate post-treatment motor symptoms according to stimulation pattern and stimulation target. **(A)** iTBS in the OFF medication state; **(B)** iTBS in the ON medication state.

Additional subgroup analyses according to stimulation target did not demonstrate statistically significant differences between subgroups. These findings should be interpreted cautiously because several subgroups contained a limited number of studies.

##### 1-month follow-up

Five studies (27, 28, 31, 34, 35) evaluated the 1-month follow-up effects of transcranial TBS on motor outcomes (Figure 4),. No statistically significant benefit was observed in either the OFF or ON medication states. The OFF medication subgroup showed moderate heterogeneity (*I*^2^ = 59%), although interpretation was limited by the inclusion of only two studies, preventing exploration of potential sources of heterogeneity or sensitivity analyses. No evidence of statistical heterogeneity was observed in the ON medication subgroup.

**Figure 4:**
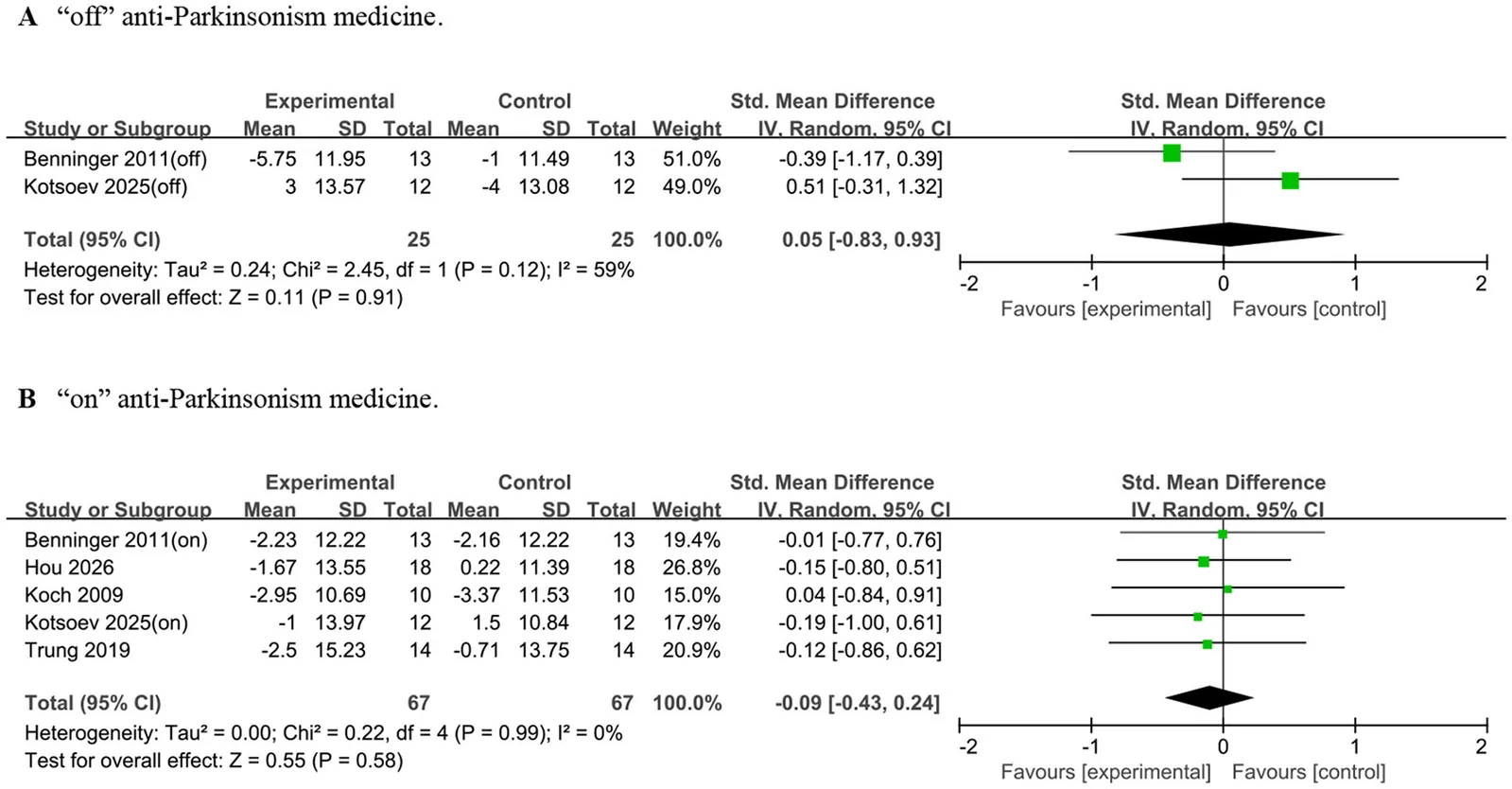
Forest plots of the effects of transcranial TBS on motor symptoms at 1-month follow-up assessed by UPDRS-III/MDS-UPDRS-III. **(A)** OFF medication state; **(B)** ON medication state.

#### Effects of transspinal TBS on motor symptoms

Two studies (17, 37) evaluating transspinal TBS assessed immediate post-treatment motor symptoms using UPDRS-III in the ON medication state. cTBS was applied at the midline over the seventh cervical vertebra (17), whereas iTBS was applied at the midline over the third thoracic vertebra (37). Compared with sham stimulation, active transspinal TBS was associated with improved motor symptoms (Figure 5) (*MD* = −8.39; 95% *CI*, −14.89 to −1.88; *Z* = 2.53; *P* = 0.01), indicating a clinically meaningful improvement (39, 40). No evidence of statistical heterogeneity was detected (χ^2^ = 0.46; *P* = 0.50; *I*^2^ = 0%).

**Figure 5:**
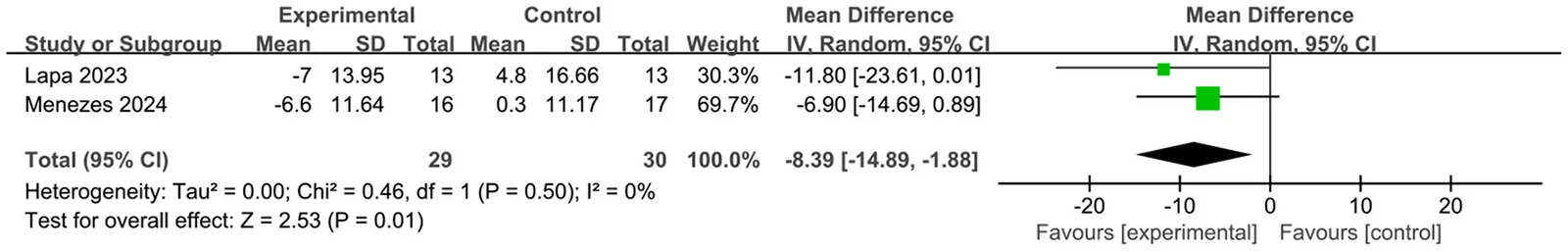
Forest plot of the effects of transspinal TBS on immediate post-treatment motor symptoms assessed by UPDRS-III in the ON medication state.

#### Effects of transcranial TBS on gait performance

Three studies evaluated gait performance using the 10-meter walk test (10WMT) (27, 28) or 20-meter walk test (20WMT) (30) in the OFF medication state. iTBS was delivered to the bilateral M1 (27) or the bilateral DLPFC and M1 (28), whereas cTBS was delivered to the left SMA (30). Compared with sham stimulation, the pooled estimate did not reach statistical significance for gait performance (Figure 6) (*SMD* = −0.30; 95% *CI*, −0.71 to 0.12; *Z* = 1.41; *P* = 0.16). No evidence of statistical heterogeneity was detected (χ^2^ = 0.85; *P* = 0.66; *I*^2^ = 0%).

**Figure 6:**
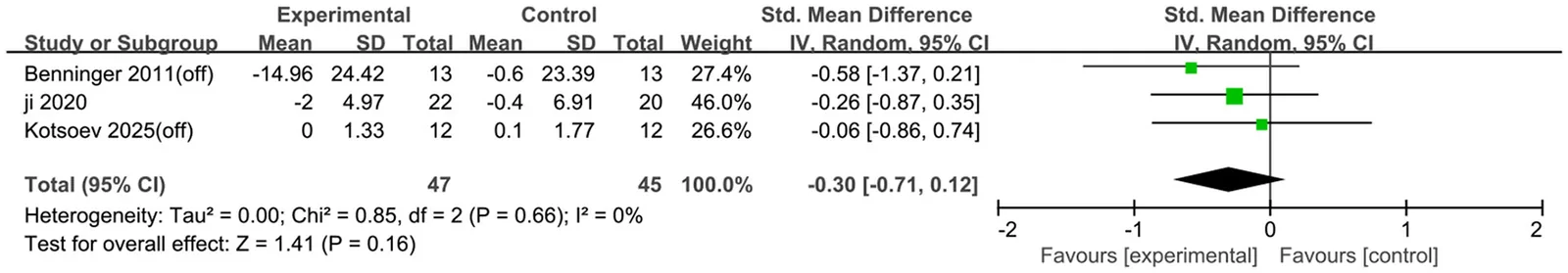
Forest plot of the effects of transcranial TBS on immediate post-treatment gait performance assessed by 10WMT/20WMT in the OFF medication state.

#### Effects of transcranial TBS on depressive symptom scores

##### Immediate post-treatment

Four studies evaluating transcranial TBS assessed depressive symptom severity at the immediate post-treatment assessment using the Beck Depression Inventory (BDI) (28, 32, 34) or the 24-item Hamilton Depression Rating Scale (HAMD-24) (35). iTBS was delivered to the bilateral DLPFC and M1 in one study (28), whereas the left DLPFC was targeted in the other three studies (32, 34, 35). One study (35) specifically recruited patients with mild depressive symptoms, whereas the remaining studies did not select participants based on clinically relevant depressive symptoms at baseline. Compared with sham stimulation, active transcranial iTBS was associated with a reduction in depressive symptom scores (Figure 7; *SMD* = −0.60; 95% *CI*, −1.07 to −0.12; *Z* = 2.47; *P* = 0.01), indicating a moderate standardized effect size (41). The analysis showed low-to-moderate heterogeneity that was not statistically significant (χ^2^ = 4.72; *P* = 0.19; *I*^2^ = 36%).

**Figure 7:**
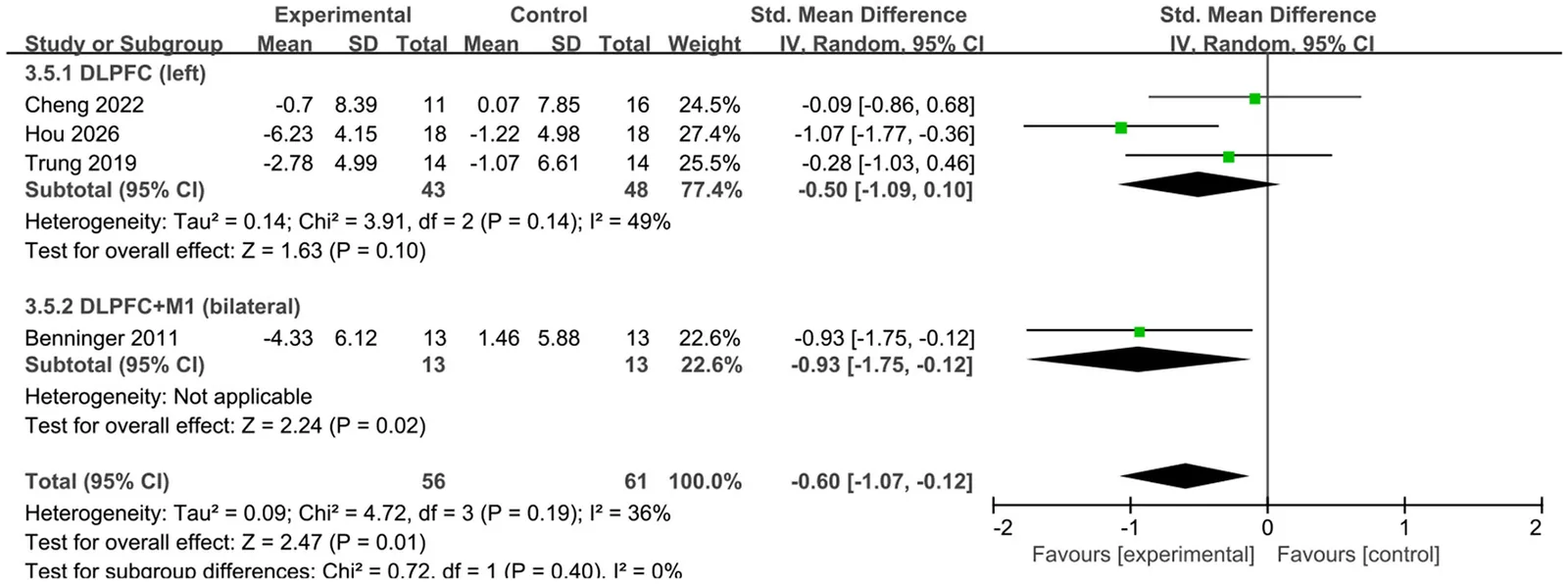
Forest plots of the effects of transcranial iTBS on depressive symptom scores at immediate post-treatment and subgroup analyses according to stimulation target.

Subgroup analyses according to stimulation target did not demonstrate statistically significant differences between subgroups (Figure 7). The DLPFC subgroup showed relatively higher variability (*I*^2^ = 49%), although this did not exceed the predefined threshold for substantial heterogeneity. The DLPFC+M1 subgroup included only one study and therefore could not be assessed for heterogeneity.

One study (17) evaluating transspinal TBS reported depressive symptom outcomes using the Hospital Anxiety and Depression Scale–Depression subscale (HADS-D) at the immediate post-treatment assessment; however, it was not included in the quantitative synthesis because only one study was available.

##### 1-month follow-up

Three studies (28, 34, 35) evaluated depressive symptom changes at the 1-month follow-up after transcranial iTBS (Figure 8). The pooled estimate did not reach statistical significance. Moderate heterogeneity was observed, which may be related to differences in depression assessment instruments (BDI and HAMD-24). Sensitivity analyses, including exclusion of potential sources of heterogeneity and leave-one-out analyses, did not materially change the overall findings (Figure 8B; Supplementary Figure S7).

**Figure 8:**
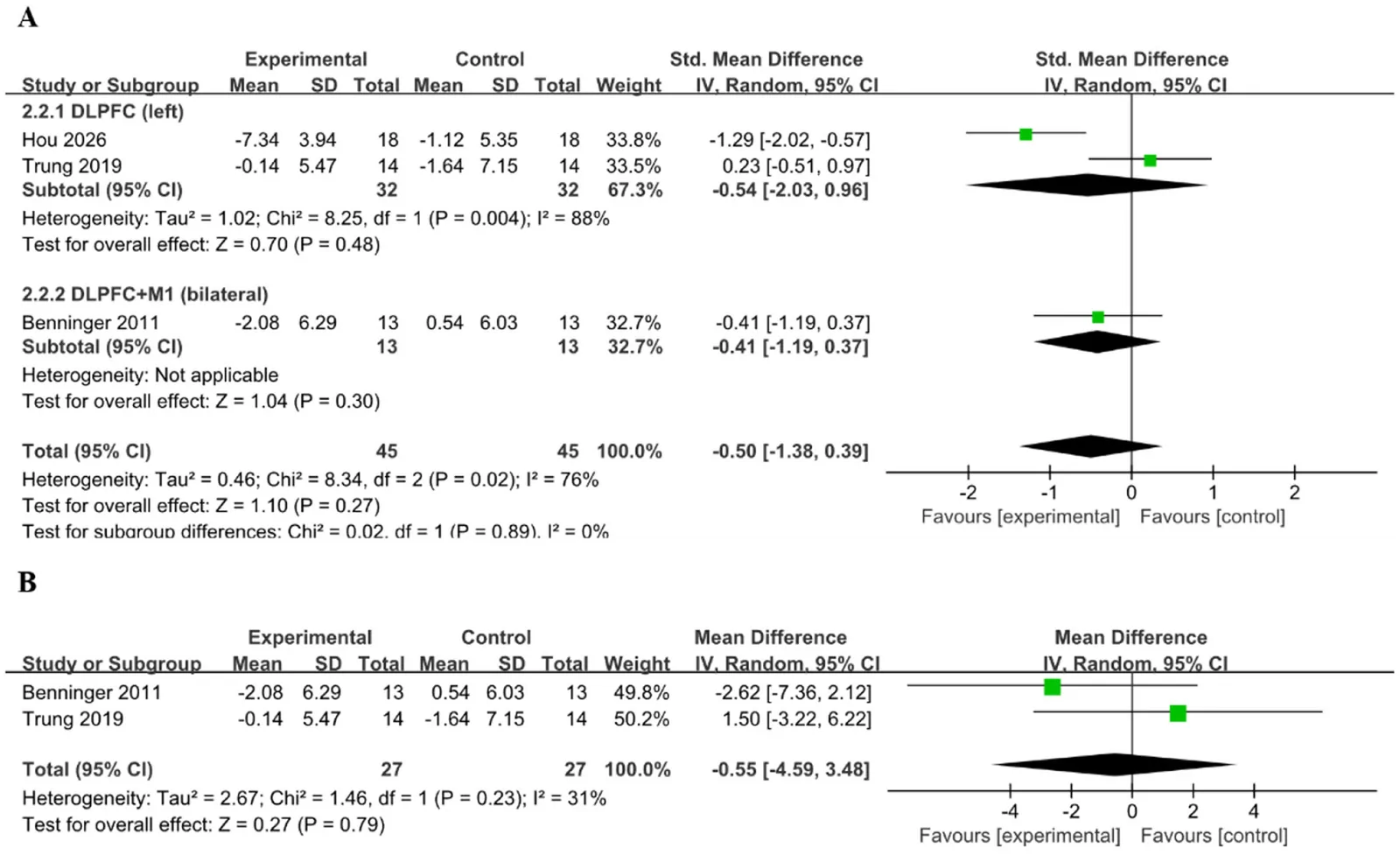
Forest plots of the effects of transcranial iTBS on depressive symptom scores at 1-month follow-up. **(A)** Main meta-analysis with subgroup analyses according to stimulation target. **(B)** Sensitivity analysis excluding potential sources of heterogeneity.

#### Effects of transcranial TBS on cognitive function

Three studies (29, 32, 33) evaluated cognitive outcomes following transcranial TBS in patients with Parkinson's disease and mild cognitive impairment (PD-MCI), with iTBS delivered to the left DLPFC in all three studies. Two studies (32, 33) assessed global cognitive function using the Montreal Cognitive Assessment (MoCA), a global cognitive screening tool covering multiple cognitive domains. In contrast, Lang et al. (29) assessed cognitive performance using composite z-scores derived from multiple neuropsychological domains, including executive function, attention, memory, language, and visuospatial ability.

Although both approaches evaluate cognitive function, MoCA and multidomain composite z-scores represent different cognitive assessment strategies and may capture different aspects of cognition. Therefore, these outcomes were considered insufficiently comparable for quantitative pooling. The composite z-score study was not included in the MoCA-based meta-analysis. Quantitative synthesis was therefore performed using MoCA-based outcomes only.

##### Immediate post-treatment cognitive outcomes

Two studies assessed cognitive function using MoCA immediately after intervention. Compared with sham stimulation, active transcranial TBS was associated with higher MoCA scores (Figure 9; *MD* = 3.20, 95% *CI*, 1.59 to 4.81; *Z* = 3.89; *P* = 0.0001), suggesting a potentially clinically relevant improvement in cognitive screening performance (42). No evidence of statistical heterogeneity was detected (χ^2^ = 0.07; *P* = 0.79; *I*^2^ = 0%).

**Figure 9:**
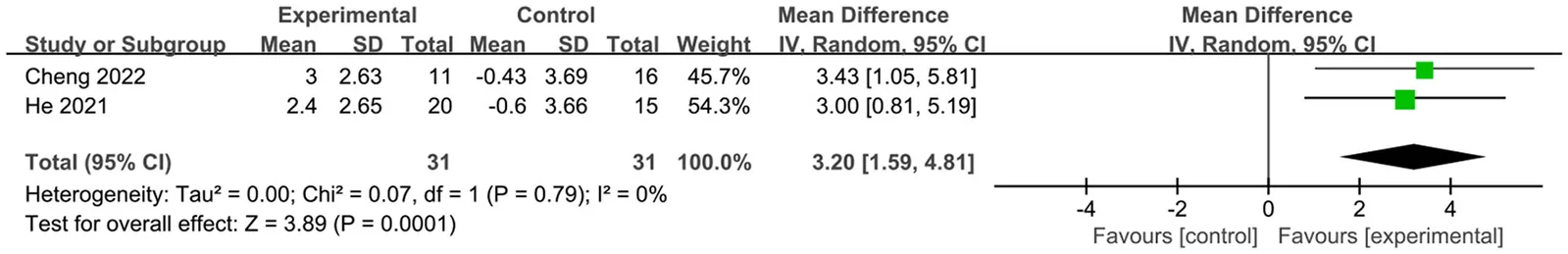
Forest plot of the effects of transcranial TBS on MoCA scores at immediate post-treatment.

##### Three-month follow-up cognitive outcomes

Two studies evaluated cognitive outcomes using MoCA at the 3-month follow-up. Compared with sham stimulation, active transcranial TBS was associated with higher MoCA scores (Figure 10; *MD* = 2.51, 95% *CI*, 0.87 to 4.16; *Z* = 2.99; *P* = 0.003), suggesting a potentially clinically relevant improvement in cognitive screening performance (42). No evidence of statistical heterogeneity was detected (χ^2^ = 0.05; *P* = 0.82; *I*^2^ = 0%).Given the limited number of included studies and participants, these MoCA-specific findings should be interpreted cautiously and considered as preliminary evidence requiring confirmation in larger randomized controlled trials.

**Figure 10:**
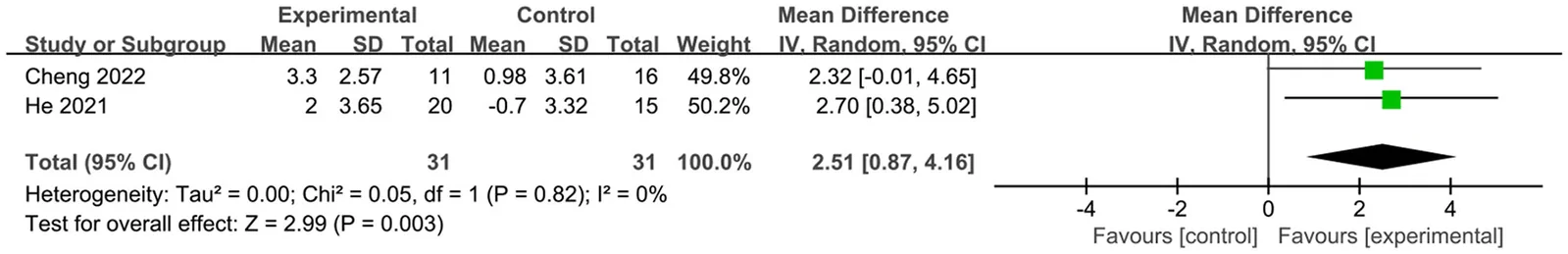
Forest plot of the effects of transcranial TBS on MoCA scores at 3-month follow-up.

#### Safety and adverse events

Safety outcomes were summarized separately according to stimulation site because adverse event profiles may differ between transcranial and transspinal TBS.

Among studies applying active transcranial TBS, two studies (29, 34) involving 35 participants did not report adverse events, whereas three studies (30, 31, 33) involving 52 participants reported that no adverse events occurred following stimulation. The remaining five studies (27, 28, 32, 35, 36), involving 79 participants receiving active transcranial TBS, provided detailed information on specific adverse events and their frequencies (Table 3). Reported adverse events included sleepiness, headache, attention span deficiency, pre-syncope, localized pain/discomfort (including neck pain), dizziness, tinnitus, and tenderness at the stimulation site.

**Table 3:** Summary of adverse events reported in patients receiving active transcranial TBS.

Study	Adverse events during session	Incidence
Kotsoev et al. (27)	Sleepiness	10/12 (83.3%)
	Headache	3/12 (25%)
	Attention span deficiency	2/12 (16.7%)
	Pre-syncope	2/12 (16.7%)
	Other localized pain (neck)	1/12 (8.3%)
	Dizziness	1/12 (8.3%)
Benninger et al. (28)	Other localized pain/discomfort	9/13 (69.2%)
	Tinnitus	1/13 (7.7%)
Cheng et al. (32)	Headache	1/11 (9.1%)
Hou et al. (35)	Tenderness at stimulation site	1/18 (5.6%)
Zhang et al. (36)	Headache	1/25 (4%)

Two studies evaluated safety outcomes following active transspinal TBS (17, 37), involving 29 participants (Table 4). Reported adverse events included drowsiness, insomnia, urinary incontinence, dizziness, headache, and transient paresthesia. Urinary incontinence and transient paresthesia were reported only in studies applying transspinal TBS.

**Table 4:** Summary of adverse events reported in patients receiving active transspinal TBS.

Study	Adverse events during session	Incidence
Menezes et al. (37)	Drowsiness, insomnia, urinary incontinence	3/16 (18.8%)
Lapa et al. (17)	Dizziness	1/13 (7.7%)
	Headache	1/13 (7.7%)
	Transient paresthesia	1/13 (7.7%)

#### Certainty of evidence

The certainty of evidence varied across outcomes. High-certainty evidence supported the effects of transcranial TBS on motor symptoms in the OFF-medication state immediately after treatment and on depressive symptoms immediately after treatment. Moderate-certainty evidence supported the effects of transcranial TBS on motor symptoms in the ON-medication state immediately after treatment, the potential benefits of transspinal TBS on motor symptoms in the ON-medication state immediately after treatment, and the effects of transcranial TBS on cognitive function immediately after treatment and at 3-month follow-up. Low-certainty evidence was observed for motor symptoms in the ON-medication state at 1-month follow-up and gait performance in the OFF-medication state immediately after treatment, while very low-certainty evidence was observed for motor symptoms in the OFF-medication state at 1-month follow-up and depressive symptoms at 1-month follow-up. The main reasons for downgrading the certainty of evidence were risk of bias, inconsistency, and imprecision, particularly where confidence intervals crossed the line of no effect or the available information was limited.

## Discussion

Compared with the previous meta-analysis published in 2022 (3), this updated review incorporated newly available randomized controlled trials and expanded the evidence base to include both transcranial and transspinal TBS. Importantly, the pooled findings should be interpreted in the context of substantial heterogeneity in anatomical stimulation targets and stimulation protocols across both transcranial and transspinal TBS studies. Among transcranial TBS studies, different stimulation targets may engage distinct functional brain networks, while differences in transspinal stimulation sites may preferentially engage distinct spinal afferent and motor pathways. High-certainty evidence supports short-term motor benefits of transcranial TBS in the OFF-medication state and reductions in depressive symptom scores. Evidence for cognitive benefits and transspinal TBS was of moderate certainty, although these findings were based on a limited number of trials and warrant confirmation in larger randomized controlled trials.

For motor symptoms, high-certainty evidence suggests that transcranial TBS may improve UPDRS-III/MDS-UPDRS-III scores in the OFF-medication state immediately after treatment, whereas moderate-certainty evidence showed no clear benefit in the ON-medication state. Previous studies (3, 4) have similarly suggested that motor effects of non-invasive brain stimulation may be more readily detected after withdrawal of dopaminergic medication. One possible explanation (43) is that dopaminergic medication may reduce the measurable additional benefit of neuromodulation by alleviating motor symptoms, whereas the OFF state may provide a greater opportunity to detect stimulation-related changes. Furthermore, acute non-physiological dopamine surges during disease progression may negatively influence cortical plasticity and potentially affect responsiveness to TBS (44). However, these findings should be interpreted cautiously because OFF and ON medication analyses were based on different sets of studies rather than direct within-subject comparisons. Although the pooled effect size was moderate (38), the clinical magnitude of this improvement remains difficult to determine because SMD values cannot be directly translated into UPDRS-III/MDS-UPDRS-III point changes.

The therapeutic response to TBS is likely influenced by multiple interacting factors beyond stimulation parameters. Previous studies (38, 44, 45) have suggested that treatment response to rTMS in Parkinson's disease may vary according to disease characteristics, treatment protocols, and individual neuroplastic responses. However, these factors could not be fully explored in the present meta-analysis because of the limited number of available trials and insufficient information for further stratified analyses. Among clinically available factors, subgroup analyses did not identify significant differences according to stimulation protocol or target site, although these findings should be interpreted cautiously given the small number of studies in several comparisons. Mechanistically, stimulation of regions such as the primary motor cortex or supplementary motor area may influence dysfunctional cortico–basal ganglia–thalamo–cortical circuits involved in motor initiation and execution (46–48); however, the optimal stimulation strategy for Parkinson's disease remains uncertain.

Regarding the durability of motor effects, high-certainty evidence supports short-term motor benefits of transcranial TBS in the OFF-medication state, whereas evidence for sustained effects at 1-month follow-up remains very uncertain. Previous studies (4, 49–52) have similarly suggested that non-invasive brain stimulation may induce short-term modulation of motor symptoms, while the persistence of clinical benefits remains unclear. One possible explanation (4, 50, 53, 54) is that transcranial TBS may produce transient changes in cortical excitability and motor network activity, whereas repeated stimulation or longer treatment courses may be required to achieve more durable neuroplastic effects in the context of progressive neurodegeneration. Future studies with larger samples and longer follow-up are needed to clarify the durability of TBS effects.

Gait outcomes did not show clear improvement following transcranial TBS. However, this finding should be interpreted cautiously because the evidence was derived from only three small trials and was rated as low certainty.

Notably, this updated review identified emerging evidence for transspinal TBS, an approach that has been rarely evaluated in previous evidence syntheses. Both included transspinal TBS studies assessed motor outcomes in the ON medication state. Moderate-certainty evidence suggests that active transspinal TBS may provide motor benefits compared with sham stimulation. however, these findings were based on only two randomized controlled trials with small sample sizes and should therefore be interpreted cautiously. As an emerging neuromodulation approach, transspinal TBS requires further validation through larger randomized controlled trials with longer follow-up to determine its efficacy, durability, and clinical relevance in Parkinson's disease.

For depressive symptoms, high-certainty evidence suggests that transcranial TBS may reduce depressive symptom scores in individuals with Parkinson's disease. However, most included studies did not specifically enroll patients with clinically diagnosed depression, with only one study (35) including patients with mild depressive symptoms. Therefore, the applicability of these findings to PD patients with comorbid depression remains uncertain. Furthermore, although the pooled effect indicated a moderate standardized reduction in depressive symptom scores (41), its clinical relevance remains uncertain because SMD values cannot be directly interpreted as changes on individual depression scales.

Most studies assessing depressive outcomes involved DLPFC stimulation, either alone or combined with motor cortex stimulation. Although the DLPFC-targeted subgroup did not reach statistical significance, this may be related to the limited number of studies. Neuroimaging studies have demonstrated functional and structural alterations in pre-frontal regions in depressed PD patients, including reduced DLPFC resting-state activity associated with depressive symptom severity and decreased left pre-frontal cortical thickness linked to depression in PD (55, 56).These findings suggest that DLPFC-related network dysfunction may contribute to depressive symptoms in PD, and modulation of these networks by DLPFC-targeted TBS may represent a potential mechanism underlying reductions in depressive symptom scores. However, in studies combining DLPFC and motor cortex stimulation, the contribution of concurrent motor improvement to changes in depressive symptom scores cannot be excluded.

For transspinal TBS, depressive outcomes were reported in only one study and were therefore not included in quantitative synthesis. Given that transspinal stimulation primarily targets spinal circuits rather than networks involved in emotional regulation, changes in depressive symptom scores should be interpreted cautiously and may be influenced by improvements in motor function.

Regarding cognitive outcomes, moderate-certainty evidence suggests that DLPFC-targeted iTBS may improve MoCA scores in individuals with Parkinson's disease and mild cognitive impairment. Both included studies enrolled patients with PD-related mild cognitive impairment and applied DLPFC-targeted iTBS. The DLPFC is a key component of pre-frontal cognitive-control networks involved in executive processes, working memory, and goal-directed behavior (57–60). Reduced mid-frontal theta activity during cognitive control tasks has been reported in Parkinson's disease, suggesting impaired recruitment of these networks (61), whereas DLPFC-targeted iTBS has been shown to enhance theta power within DLPFC-related networks (62). These findings provide a potential neurophysiological basis for the observed cognitive effects of DLPFC-targeted iTBS. However, the evidence remains limited because it is based on only two randomized controlled trials with small sample sizes.

Given that the available evidence mainly supports short-term effects, TBS should currently be considered an adjunct to optimized pharmacological and rehabilitative management rather than a replacement for conventional therapy. The relatively brief stimulation duration of TBS compared with conventional rTMS protocols may offer practical advantages for future clinical implementation. Some included studies combined TBS with rehabilitation approaches, suggesting its potential role within multimodal treatment strategies for Parkinson's disease. High-certainty evidence supports short-term improvements in motor symptoms and depressive symptom scores, whereas evidence for cognitive benefits is of moderate certainty and warrants further confirmation in larger randomized controlled trials. Overall, patients with persistent motor or non-motor symptoms despite optimized medical therapy may represent potential candidates for future TBS-based interventions.

### Strengths and limitations

This review has several strengths. First, this updated systematic review and meta-analysis incorporated recently available randomized controlled trials and provided a comprehensive evaluation of TBS effects on both motor and non-motor symptoms in Parkinson's disease. Second, this review included emerging evidence on transspinal TBS, an approach that has received limited evaluation in previous evidence syntheses. Third, both immediate and follow-up outcomes were assessed when data were available, allowing evaluation of the short-term efficacy and durability of treatment effects. Finally, the certainty of evidence was systematically evaluated using the GRADE framework (Table 5).

**Table 5:** Summary of findings and GRADE certainty assessment for theta burst stimulation in Parkinson's disease.

Certainty assessment							No. of patients		Effect	Certainty	Importance
No. of studies	Study design	Risk of bias	Inconsistency	Indirectness	Imprecision	Other considerations	Theta Burst Stimulation	Sham Stimulation	Absolute (95% CI)		
Motor symptoms (transcranial TBS, OFF medication state; immediate post-treatment) (assessed with: UPDRS-III/MDS-UPDRS-III)											
4	Randomized trials	Not serious	Not serious	Not serious	Not serious	None	72	70	SMD **0.43 SD lower** (0.78 lower to 0.09 lower)	⊕⊕⊕⊕ High	CRITICAL
Motor symptoms (transcranial TBS, OFF medication state) (follow-up: 1 month; assessed with: UPDRS-III/MDS-UPDRS-III)											
2	Randomized trials	Serious^a^	Serious^b^	Not serious	Very serious^c^	None	25	25	SMD **0.05 SD higher** (0.83 lower to 0.93 higher)	⊕◯◯◯ Very low^a, b, c^	CRITICAL
Motor symptoms (transcranial TBS, ON medication state; immediate post-treatment) (assessed with: UPDRS-III/MDS-UPDRS-III)											
6	Randomized trials	Not serious	Not serious	Not serious	Serious^d^	None	92	92	SMD **0.07 SD lower** (0.36 lower to 0.22 higher)	⊕⊕⊕◯ Moderate^d^	CRITICAL
Motor symptoms (transcranial TBS, ON medication state) (follow-up: 1 month; assessed with: UPDRS-III/MDS-UPDRS-III)											
5	Randomized trials	serious^a^	Not serious	Not serious	Serious^d^	None	67	67	SMD **0.09 SD lower** (0.43 lower to 0.24 higher)	⊕⊕◯◯ Low^a, d^	CRITICAL
Motor symptoms (transspinal TBS, ON medication state; immediate post-treatment) (assessed with: UPDRS-III)											
2	Randomized trials	Not serious	Not serious	Not serious	Serious^e^	None	29	30	MD **8.39 lower** (14.89 lower to 1.88 lower)	⊕⊕⊕◯ Moderate^e^	CRITICAL
Gait performance (transcranial TBS, OFF medication state; immediate post-treatment) (assessed with: 10WMT/20WMT)											
3	Randomized trials	Not serious	Not serious	Not serious	Very serious^c^	None	47	45	SMD **0.3 SD lower** (0.71 lower to 0.12 higher)	⊕⊕◯◯ Low^c^	IMPORTANT
Depressive symptoms (transcranial TBS; immediate post-treatment) (assessed with: BDI/HAMD-24)											
4	Randomized trials	Not serious	Not serious	Not serious	Not serious	None	56	61	SMD **0.6 SD lower** (1.07 lower to 0.12 lower)	⊕⊕⊕⊕ High	IMPORTANT
Depressive symptoms (transcranial TBS) (follow-up: 1 month; assessed with: BDI/HAMD-24)											
3	Randomized trials	Not serious	Serious^f^	Not serious	Very serious^c^	None	45	45	SMD **0.5 SD lower** (1.38 lower to 0.39 higher)	⊕◯◯◯ Very low^c, f^	IMPORTANT
Cognitive function (transcranial TBS; immediate post-treatment) (assessed with: MoCA)											
2	Randomized trials	Not serious	Not serious	Not serious	Serious^e^	None	31	31	MD **3.2 higher** (1.59 higher to 4.81 higher)	⊕⊕⊕◯ Moderate^e^	IMPORTANT
Cognitive function (transcranial TBS) (follow-up: 3 months; assessed with: MoCA)											
2	Randomized trials	Not serious	Not serious	Not serious	Serious^e^	None	31	31	MD **2.51 higher** (0.87 higher to 4.16 higher)	⊕⊕⊕◯ Moderate^e^	IMPORTANT

CI, confidence interval; MD, mean difference; SMD, standardized mean difference.

^a^Downgraded by one level for risk of bias because one included trial had a high risk of bias due to missing outcome data at follow-up, with differential loss to follow-up and potential for missingness to be related to participants' clinical status.

^b^Downgraded by one level for inconsistency because moderate statistical heterogeneity was observed between the included trials (*I*^2^ = 59%), and the small number of studies limited the ability to identify potential sources of variability.

^c^Downgraded by two levels for very serious imprecision because the confidence interval crossed the line of no effect and indicated substantial uncertainty regarding the magnitude and direction of the treatment effect, with limited overall information.

^d^Downgraded by one level for imprecision because the confidence interval crossed the line of no effect, indicating uncertainty regarding the presence and magnitude of a treatment effect.

^e^Downgraded by one level for imprecision because the total information size was limited, resulting in uncertainty in the precision of the pooled estimate.

^f^Downgraded by one level for inconsistency because substantial heterogeneity was observed across the included trials (*I*^2^ = 76%), suggesting variability in the estimated treatment effects.

Several limitations should be acknowledged. First, randomized crossover trials were excluded because of concerns related to the progressive nature of Parkinson's disease, potential carry-over effects associated with washout periods, and the lack of within-participant data required for appropriate paired analyses. Although these trials were identified during full-text assessment, their exclusion may have reduced the comprehensiveness of the available randomized evidence and should be considered when interpreting the findings. Second, although the number of available randomized trials has increased, the overall evidence base remains limited, with relatively small sample sizes and insufficient reporting of clinical and neurophysiological characteristics. These limitations constrained subgroup analyses and prevented reliable evaluation of potential moderators, such as disease stage and individual neuroplastic capacity. Third, evidence for some non-motor outcomes, particularly cognitive outcomes, was based on a small number of trials and should therefore be interpreted cautiously. In addition, the limited availability of follow-up data prevented firm conclusions regarding the durability of treatment effects. Finally, evidence regarding transspinal TBS remains preliminary because only two randomized trials were available. Moreover, substantial heterogeneity in anatomical stimulation targets and stimulation protocols should be considered when interpreting the pooled findings. Transcranial TBS studies targeted different brain regions that may engage distinct functional brain networks, while the two transspinal TBS studies differed in stimulation sites and stimulation patterns, which may differentially recruit spinal afferent and motor pathways. These sources of heterogeneity may contribute to variability in clinical effects and warrant further investigation in larger, adequately controlled studies.

## Conclusion

In conclusion, TBS represents a promising adjunctive neuromodulation approach for Parkinson's disease. High-certainty evidence suggests that transcranial TBS may provide short-term motor benefits in the OFF-medication state and may reduce depressive symptom scores. Moderate-certainty evidence suggests potential cognitive benefits following transcranial TBS and motor benefits following transspinal TBS, although these findings are based on a limited number of trials and should be interpreted cautiously. The heterogeneity in anatomical stimulation targets and stimulation protocols further limits the interpretation of pooled effects. Larger randomized controlled trials with standardized stimulation protocols and longer follow-up are needed to clarify the durability and clinical relevance of these effects and to identify patients most likely to benefit from TBS.

## Data Availability

The datasets generated and/or analysed during the current study are available from the corresponding author on reasonable request.
