# Supplementary material for: Effectiveness and safety of theta burst stimulation for Parkinson's disease: an updated systematic review and meta-analysis of randomized parallel-group controlled trials
Source: Front Neurol. 2026 Sep 23;17:1915632. doi: 10.3389/fneur.2026.1915632 (PMC13646720; doi:10.3389/fneur.2026.1915632)
Supplement: Supplementary file 1 [file Supplementary_File_1.docx]

Supplementary Figures S1.Risk-of-bias assessment for immediate motor outcomes.

Supplementary Figures S2. Risk-of-bias assessment for 1-month follow-up motor outcomes.

Supplementary Figures S3. Risk-of-bias assessment for gait outcomes.

Supplementary Figures S4. Risk-of-bias assessment for immediate depressive outcomes.

Supplementary Figures S5. Risk-of-bias assessment for 1-month follow-up depressive outcomes.

Supplementary Figures S6. Risk-of-bias assessment for cognitive function outcomes (immediate post-treatment and 3-month follow-up).The RoB 2 judgments were identical for both assessment time points; therefore, a single risk-of-bias assessment is presented.

Supplementary Figure S7. Leave-one-out sensitivity analyses for the effects of transcranial iTBS on depressive symptom scores at 1-month follow-up.

(A) Exclusion of Hou et al.; (B) Exclusion of Trung et al.; (C) Exclusion of Benninger et al.

Supplementary Table S1. Complete search strategies for all databases.

| Database | date | Search strategy | Records |
| --- | --- | --- | --- |
| PubMed | 2026/7/28 | ((("Parkinson Disease"[Mesh]) OR (Parkinson Disease[Title/Abstract] OR Idiopathic Parkinson Disease[Title/Abstract] OR Idiopathic Parkinson's Disease[Title/Abstract] OR Lewy Body Parkinson Disease[Title/Abstract] OR Lewy Body Parkinson's Disease[Title/Abstract] OR Paralysis Agitans[Title/Abstract] OR Parkinson Disease, Idiopathic[Title/Abstract] OR Parkinson's Disease[Title/Abstract] OR Parkinson's Disease, Idiopathic[Title/Abstract] OR Parkinson's Disease, Lewy Body[Title/Abstract] OR Primary Parkinsonism[Title/Abstract] OR Parkinsonism, Primary[Title/Abstract])) AND (theta-burst stimulation[Title/Abstract] OR TBS[Title/Abstract] OR repetitive transcranial magnetic stimulation[Title/Abstract] OR rTMS[Title/Abstract] OR Trans-Spinal Theta Burst Magnetic Stimulation[Title/Abstract] OR TsMS[Title/Abstract] OR non-invasive stimulation[Title/Abstract])) AND (randomized controlled trial[Publication Type] OR randomized[Title/Abstract] OR placebo[Title/Abstract]) | 208 |
| Embase | 2026/7/28 | #1. 'parkinson disease'/exp #2. ''idiopathic parkinsonism':ti,ab,kw OR 'lewy odieof parkinson disease':ti,ab,kw OR 'lewy bodies of parkinsons disease':ti,ab,kw OR 'lewy body parkinson disease':ti,ab,kw OR 'lewy body parkinsons disease':ti,ab,kw OR 'paralysis agitans':ti,ab,kw OR 'parkinson dementia complex':ti,ab,kw OR 'parkinsons disease':ti,ab,kw OR 'primary parkinsonism':ti,ab,kw OR 'parkinson disease':ti,ab,kw #3. 'theta-burst stimulation':ti,ab,kw ORtbs:ti,ab,kw OR 'repetitive transcranial magnetic stimulation':ti,ab,kw OR rtms:ti,ab,kw OR 'trans-spinal theta burst magnetic stimulation':ti,ab,kw OR tsms:ti,ab,kw OR 'non-invasive stimulation':ti,ab,kw #4. random:ti,ab,kw OR placebo:ti,ab,kw OR 'doubleblind':ti,ab,kw #5. #1 OR #2 #6. #3 AND #4 AND #5 | 277 |
| Cochrane | 2026/7/28 | #1 MeSH descriptor: [Parkinson Disease] explode all trees #2 (Lewy Body Parkinson's Disease):ti,ab,kw OR (Idiopathic Parkinson's Disease):ti,ab,kw OR (Parkinson Disease, Idiopathic):ti,ab,kw OR (Parkinson's Disease):ti,ab,kw OR (Primary Parkinsonism):ti,ab,kw OR (Parkinson's Disease, Lewy Body):ti,ab,kw OR (Paralysis Agitans):ti,ab,kw OR (Parkinson's Disease, Idiopathic):ti,ab,kw OR (Lewy Body Parkinson Disease):ti,ab,kw OR (Idiopathic Parkinson Disease):ti,ab,kw OR (Parkinsonism, Primary):ti,ab,kw #3 (theta-burst stimulation):ti,ab,kw OR (TBS):ti,ab,kw OR (repetitive transcranial magnetic stimulation):ti,ab,kw OR (rTMS):ti,ab,kw OR (Trans-Spinal Theta Burst Magnetic Stimulation):ti,ab,kw OR (TsMS):ti,ab,kw OR (non-invasive stimulation):ti,ab,kw #4 #1 OR #2 #5 #3 AND #4 | 695 |
| Web of Science | 2026/7/28 | ((TS=(Parkinson Disease OR Idiopathic Parkinson Disease OR Idiopathic Parkinson's Disease OR Lewy Body Parkinson Disease OR Lewy Body Parkinson's Disease OR Paralysis Agitans OR Parkinson Disease, Idiopathic OR Parkinson's Disease OR Parkinson's Disease, Idiopathic OR Parkinson's Disease, Lewy Body OR Primary Parkinsonism OR Parkinsonism, Primary)) AND TS=(theta-burst stimulation OR TBS OR repetitive transcranial magnetic stimulation OR rTMS OR Trans-Spinal Theta Burst Magnetic Stimulation OR TsMS OR non-invasive stimulation)) AND TS=(randomized controlled trial OR random* OR placebo) | 493 |
| ClinicalTrials.gov | 2026/7/28 | AREA[ConditionSearch](Parkinson disease) AND AREA[InterventionSearch](theta-burst stimulation OR trans-spinal theta burst magnetic stimulation OR TsMS ) | 35 |

Supplementary Table 2. Key studies excluded after full-text assessment.This table summarizes key studies excluded based on study-design or comparator criteria and does not include studies excluded because of ineligible stimulation types.

| Study | Study design | Primary reason for exclusion |
| --- | --- | --- |
| Chen, 2025 | Non-randomized study | Non-randomized design |
| Sun, 2024 | Open-label trial | Non-randomized design |
| Gouriou, 2024 | Single-case report | Non-randomized design |
| Rashid-Lopez, 2023 | Single-case report | Non-randomized design |
| Pastore-Wapp, 2023 | Pilot feasibility study | Non-randomized design |
| Degardin, 2012 | Non-randomized study | Non-randomized design |
| Wu, 2025 | Randomized parallel-group trial | No sham control; iTBS vs. HF-rTMS |
| Liu, 2025 | Randomized parallel-group trial | No sham control; iTBS vs. HF-rTMS |
| Kishore, 2011 | Randomized parallel-group trial | No sham control; iTBS vs. cTBS |
| Shi, 2025 | Randomized crossover trial | Crossover design |
| Eggers, 2015 | Randomized crossover trial | Crossover design |
| Potvin-Desrochers, 2023 | Randomized crossover trial | Crossover design |
| Hill, 2019 | Randomized crossover trial | Crossover design |
| Tard, 2016 | Randomized crossover trial | Crossover design |
| Bologna, 2015 | Randomized crossover trial | Crossover design |
| Macías-García, 2026 | Randomized crossover trial | Crossover design |
| Rashid-López, 2026 | Randomized crossover trial | Crossover design |
